# Outcomes of Unicompartmental Knee Arthroplasty in Patients Receiving Glucagon-like Peptide 1 Agonist Therapy: A Matched Cohort Study

**DOI:** 10.1016/j.artd.2026.101958

**Published:** 2026-02-09

**Authors:** Kevin Y. Heo, Jacob A. Worden, Emilio Arellano, Jerry Chang, Bailey J. Ross, Anthony L. Karzon, Ajay Premkumar, Jacob M. Wilson

**Affiliations:** a Department of Orthopaedic Surgery, Emory University School of Medicine, Atlanta, GA, USA; b Department of Orthopaedic Surgery, Medical College of Georgia, Augusta, GA, USA

**Keywords:** GLP-1 receptor agonist, Type 2 diabetes mellitus, Obesity, Unicompartmental knee arthroplasty, Surgical complications

## Abstract

**Background:**

Unicompartmental knee arthroplasty (UKA) has become increasingly utilized to treat single-compartment osteoarthritis, including in patients with type 2 diabetes mellitus (T2DM). Glucagon-like peptide-1 (GLP-1) agonists have revolutionized T2DM management and are also widely prescribed for managing obesity. Limited data currently exist on their impact on UKA outcomes. Therefore, this study investigated UKA outcomes in patients receiving GLP-1 agonists.

**Methods:**

Adult patients with T2DM and/or obesity undergoing UKA between 2009 and 2022 were identified utilizing an administrative claims database. Patients who were prescribed GLP-1 agonists within 6 months before surgery were 1:4 propensity score matched to controls based on age, sex, comorbidity burden, obesity class, and T2DM status. Multivariable logistic regressions were employed to examine 90-day and 1-year UKA outcomes between groups.

**Results:**

Of the 26,271 patients who underwent UKA, matching yielded 1018 GLP-1 agonist users and 4493 controls. There were no significant differences in 90-day surgical site infections (odds ratio [OR] 0.9; *P* = .79) or prosthetic joint infections (OR 0.7; *P* = .40). Patients on GLP-1 agonists had decreased odds of extended hospital length of stay (≥3 days) (OR 0.6; *P* < .001). There were no significant differences in complications at 1 year after surgery.

**Conclusions:**

Our study showed that patients receiving GLP-1 agonists did not have increased rates of adverse surgical outcomes following UKA; however, we could not definitively conclude that GLP-1 agonists served as a protective factor for decreasing complications. Given the expanding usage of GLP-1 agonists, prospective research is needed to delineate these potential risks or benefits.

## Introduction

Knee osteoarthritis (OA) is one of the most common causes of disability, leading to significant pain and impaired function [1]. Unicompartmental knee OA occurs when there is isolated degeneration of a single compartment of the knee. This may result from knee alignment disorders or from injuries that have damaged the cartilage in either compartment [2]. In patients who have failed nonoperative management, unicompartmental knee arthroplasty (UKA) provides an option for improving patient pain and function. The partial replacement allows for preservation of bone stock, faster recovery time, and reduced complications compared with total knee arthroplasty (TKA) [[3], [4], [5], [6]]. Improvements in implant technology and surgical technique have expanded the use of UKA, with excellent reported implant survivorship rates [[7], [8], [9], [10]].

Metabolic diseases play a crucial role in patients undergoing joint arthroplasty. A high body mass index (BMI > 30) has been associated with increased risk of deep infections, early revisions, and in-hospital complications in TKA patients [10,11]. In addition, type 2 diabetes mellitus (T2DM) is recognized as an independent risk factor for prosthetic joint infections (PJIs) and deep surgical site infections (SSIs) following TKA [12,13]. However, when comparing controlled and uncontrolled diabetes, the heightened risk of complications was only observed in patients who had uncontrolled diabetes, emphasizing the importance of maintaining proper glycemic control to improve patient outcomes [14].

The prevalence of T2DM in the United States rose from 9.8% to 14.3% from 1999 to 2018, with only 21.0% of these patients achieving optimal control of hemoglobin A1C [15]. Pharmacologic options for T2DM are directed toward addressing insulin resistance and decreasing pancreatic beta-cell function [16]. While metformin, sulfonylureas, and insulin have been staples of glycemic control, glucagon-like peptide-1 (GLP-1) receptor agonists and sodium-glucose cotransporter-2 inhibitors have transformed diabetes management, especially in patients undergoing orthopaedic surgery [17]. GLP-1 agonists, in particular, have demonstrated high efficacy in achieving weight loss and glycemic control [18,19]. Prior research has shown noninferior clinical outcomes following TKA in patients who have T2DM taking GLP-1 agonists [[20], [21], [22]].

While numerous studies have reported durable, effective outcomes of UKA in obese and diabetic patients [[23], [24], [25], [26]], the rapid adoption of GLP-1 agonist therapy in treating obesity and diabetes has far outpaced the literature available on outcomes, thus creating a niche into which further investigation is needed to fully elucidate how these therapeutics interplay with arthroplasty. To the authors’ knowledge, no prior studies have investigated outcomes of UKA in patients taking GLP-1 agonists. Given that UKAs are increasingly performed in patients who have severe unicompartmental OA with preservation of the remaining knee compartments, this information is valuable to have when considering surgical treatment in this population.

Therefore, the aim of this study was to compare the relative rates of complications in patients taking GLP-1 agonists compared with patients not on GLP-1 agonists following UKA while accounting for disease severity and other comorbidities. We hypothesized that patients who were on GLP-1 agonists would have no differences in postoperative complications compared with those not on these medications.

## Material and methods

### Data source

Patients were identified from the Merative MarketScan Commercial Claims and Encounters and Medicare Supplemental and Coordination of Benefit databases (Ann Arbor, MI). Institutional review board approval was not required as this study was a retrospective review of deidentified data. This database is a collection of medical insurance claims databases from over 300 employer-sponsored and Medicare supplemental plans, containing more than 240 million deidentified patient records. It provides information on inpatient admissions, outpatient visits, and pharmaceutical encounters. The authors chose to use this database because it contains data on a large number of continuously enrolled patients that allows for longitudinal follow-up. It has been successfully utilized in previous orthopaedic studies [27].

### Patient identification: inclusion and exclusion criteria

Current Procedural Terminology code “27446” (arthroplasty, knee, condyle and plateau; medial or lateral compartment) was used to identify patients over 18 years who underwent unilateral UKA from January 1, 2009, to December 31, 2022. Only patients who had a preoperative diagnosis of T2DM and/or obesity were included. Nondiabetic and nonobese patients, as well as those who had type 1 DM, were excluded from the study. Those who had a periarticular lower extremity fracture at the time of surgery were also excluded. Patients who had a history of pancreatitis or medullary thyroid cancer were excluded, as these are potential contraindications to GLP-1 agonist use [28]. International Classification of Diseases (ICD) codes and the Elixhauser method were used to identify comorbidities, including diabetes and obesity [29]. To be included in the final analysis, patients were required to be continuously enrolled within the database for at least 6 months preoperatively and 3 months postoperatively.

After the initial study population was established, patients were separated into 2 cohorts: GLP-1 agonists vs no GLP-1 agonists. Cohorts were determined utilizing prescription claims based on National Drug Codes. Per precedence, patients were included in the GLP-1 agonist cohort (treatment group) if the database recorded at least 3 fills of their GLP-1 agonist prescription within 6 months preoperatively, or if they received at least 1 fill corresponding to a 90-day or greater supply within 6 months preoperatively [21]. Patients in the no GLP-1 agonist cohort (control group) were identified as those who did not receive any GLP-1 agonists within the 6-month period before their UKA. Patients who did not meet strict criteria for the treatment or control groups were excluded from the study.

### Baseline patient information

Baseline patient demographic characteristics, diabetic prescription status, comorbidity data, and smoking status were collected. This included comorbidity status using the Elixhauser comorbidity index as previously described (grouped categorically on the basis of the number of comorbidities present), insulin status (grouped categorically as insulin dependent or not), and usage of other diabetic medications, such as metformin, sulfonylureas, thiazolidinediones, dipeptidyl peptidase 4 inhibitors, meglitinides, sodium-glucose cotransporter-2 inhibitors, and alpha-glucosidase inhibitors. As done previously by Heo et al. [21] and Wilson et al. [30], usage of other diabetic medications was grouped binomially based on the presence of additional diabetic medication usage or not. Diabetes was classified as complicated or uncomplicated based on the Elixhauser comorbidity index in order to approximate disease severity that may affect the results. Complicated diabetes is defined by the presence of end-organ damage, including peripheral neuropathy, nephropathy, peripheral artery disease, or retinopathy [31]. Obesity was subcategorized into class I/II obesity (BMI 30.0-39.9) or class III obesity (BMI > 40.0) based on ICD-9 and ICD-10 coding. Exact values for BMI or weight were not available in the database.

### Postoperative complications and health care utilization data

Postoperative resource utilization, surgical complications, and medical complications were collected for the 90-day postoperative period. These included the following utilization and complication parameters: SSI, peri-PJI, wound dehiscence, periprosthetic fracture, aseptic loosening, cardiac arrest, stroke, pneumonia, deep vein thrombosis, urinary tract infection, acute kidney injury, *Clostridium difficile* infection, hypoglycemic events, pulmonary aspiration, 90-day all-cause hospital readmission, and extended hospital length of stay (LOS) (defined as ≥3 days) [32,33]. In addition, similar to Wilson et al. [30], 1-year postoperative outcomes were collected, including SSI, wound dehiscence, PJI, periprosthetic fracture, and aseptic loosening.

### Baseline patient characteristics

*Chi*-square tests were used to determine differences in categorical comorbidity variables, and Student’s *t*-tests were used to analyze differences in continuous comorbidity variables, as indicated. In total, patients in the treatment group were younger (58.9 vs 59.4 years old, *P* = .02), more likely to be men (53.7% vs 48.0%, *P* < .001), and had a higher percentage of having at least 3 Elixhauser comorbidities compared with patients in the control group (71.1% vs 63.3%, *P* < .001) (Table 1). Furthermore, those in the treatment group had a higher prevalence of T2DM (87.8% vs 43.2%, *P* < .001), insulin-dependent diabetes (15.1% vs 1.2%, *P* < .001), complicated diabetes (37.2% vs 9.6%, *P* < .001), and usage of additional diabetic medications (55.6% vs 29.3%, *P* < .001). Patients who were in the treatment group also had a higher prevalence of congestive heart failure (7.2% vs 5.6%, *P* = .02) and class III obesity (32.8% vs 23.5%; *P* < .001); however, they had lower rates of class I/II obesity (60.2% vs 66.4%, *P* < .001). There was no difference in rates of smoking status between groups (13.8 vs 15.8, *P* = .06).

**Table 1:** Patient demographic characteristics and comorbidities: GLP-1 receptor agonist use vs no GLP-1 agonist use.

Characteristic	GLP-1 agonist	No GLP-1 agonist	*P*-value
Total (%)	1195 (4.5)	25,076 (95.5)	
Age (range)	58.9 (27-84)	59.4 (18-94)	.02
Sex, n (%)			
Men	642 (53.7)	12,038 (48.0)	<.001
Women	553 (46.3)	13,038 (52.0)	
Elixhauser, n (%)			<.001
1	126 (10.5)	3495 (13.9)	
2	220 (18.4)	5703 (22.8)	
>3	849 (71.1)	15,878 (63.3)	
T2DM, n (%)	1048 (87.8)	10,841 (43.2)	<.001
Insulin-dependent diabetes mellitus, n (%)	181 (15.1)	303 (1.2)	<.001
Other diabetes mellitus medication, n (%)	665 (55.6)	7351 (29.3)	<.001
Congestive heart failure	86 (7.2)	1414 (5.6)	.02
Complicated diabetes^a^, n (%)	445 (37.2)	2398 (9.6)	<.001
Class I/II obesity^b^, n (%)	728 (60.2)	16,694 (66.4)	<.001
Class III obesity^b^, n (%)	397 (32.8)	5909 (23.5)	<.001
Smoking status	165 (13.8)	3971 (15.8)	.06

Values are given as number of patients, with the percentage in parentheses, except for age, which is given as the mean and the range.

a Complicated diabetes, diabetes associated with end-organ damage, including peripheral neuropathy, nephropathy, peripheral artery disease, and retinopathy.

b Class I/II obesity, body mass index (30.0-39.9); class III obesity, body mass index (40.0+).

### Data analyses and propensity-score matching

In order to determine an a priori estimate of the effect size, multivariable logistic regressions were utilized for 90-day and 1-year postoperative outcomes between treatment and control groups prior to matching. The effect sizes were then obtained to determine the sample sizes to achieve a sufficient postmatch power of 0.80. Therefore, propensity-score matching was performed at a 4-to-1 ratio to control for baseline variables between cohorts with a 0.2 standard deviation caliper size. This was done to ensure the most optimal match [34]. Propensity score models included potential confounders of age, sex, Elixhauser comorbidity index (1, 2, and 3+ comorbidities), the presence of T2DM, class I/II obesity, and class III obesity.

**Figure 1:**
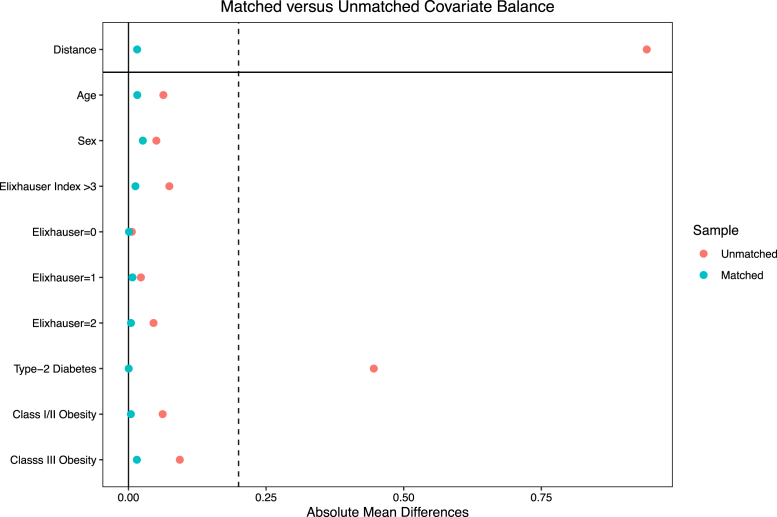
Absolute standardized mean differences of covariates in unmatched and matched datasets: GLP-1 agonist vs no GLP-1 agonist.

Propensity-score matching models resulted in a total of 5511 matched patients. Figure 1 displays the absolute standardized mean differences for all covariates utilized in the propensity-score match. All standardized mean differences for the covariates were below 0.2, indicating negligible cohort differences. Furthermore, baseline differences between cohorts were successfully balanced within the matched dataset of patients who were on GLP-1 agonists and those who were not (Table 2).

**Table 2:** Demographic characteristics and comorbidities—GLP-1 receptor agonist use vs no GLP-1 agonist use—matched dataset.

Characteristic	GLP-1 agonist	No GLP-1 agonist	*P*-value
Total (%)	1018 (18.5)	4493 (81.5)	
Age (range)	58.9 (27-84)	58.7 (18-94)	.35
Sex, n (%)			
Men	545 (53.5)	2267 (50.5)	.08
Women	473 (46.5)	2226 (49.5)	
Elixhauser, n (%)			.78
1	109 (10.7)	483 (10.7)	
2	197 (19.4)	811 (18.1)	
>3	712 (69.9)	3199 (71.2)	
T2DM, n (%)	891 (87.5)	3928 (87.4)	.83
Class I/II obesity^a^, n (%)	609 (59.8)	2654 (59.1)	.66
Class III obesity^a^, n (%)	330 (32.4)	1367 (30.4)	.21

Values are given as number of patients, with the percentage in parentheses, except for age, which is given as the mean and the range.

a Class I/II obesity, body mass index (30.0-39.9); class III obesity, body mass index (40.0+).

After propensity-score matching, multivariable logistic regressions were performed, controlling for demographics, Elixhauser comorbidity index, classes I/II and III obesity, T2DM, insulin-dependent diabetes, usage of other diabetes medications, congestive heart failure, complicated diabetes, and smoking status, to identify the effects of GLP-1 agonist use on 90-day and 1-year postoperative outcomes after UKA. All statistical analyses were conducted using R, version 4.3.0 (R Foundation for Statistical Computing, Vienna, Austria).

## Results

We identified a total of 1195 patients receiving a GLP-1 agonist and 25,076 patients not receiving a GLP-1 agonist who underwent UKA between 2009 and 2022 (Table 1). From the unmatched GLP-1 cohorts (Table 3), patients who were on GLP-1 agonists had significantly lower rates of 90-day deep vein thrombosis compared with those not on GLP-1 agonists (0.6% vs 1.3%; odds ratio [OR] 0.4; *P* = .01) as well as lower rates of extended LOS (15.1% vs 22.5%; OR 0.5; *P* < .001). However, they did have significantly higher rates of hypoglycemic events after surgery (1.7% vs 0.4%; OR 2.4; *P* = .001). At the 1-year mark, patients on GLP-1 agonists had significantly lower rates of aseptic loosening (0.5% vs 0.9%; OR 0.4; *P* = .04). There were no significant differences in rates of periprosthetic joint infections at 90 days (1.4% vs 1.4%; OR 0.9; *P* = .57) or at 1 year (1.6% vs 1.9%; OR 0.7; *P* = .14).

**Table 3:** Multivariable analyses of complications—GLP-1 agonist vs no GLP-1 agonist—all unmatched patients.

Characteristic	GLP1 agonist (%) N = 1195	No GLP-1 agonist (%) N = 25,076	OR^a^ (95% CI)	Multivariable *P*-value
90-D surgical complications				
SSI	1.8	1.4	1.0 (0.6-1.5)	.83
PJI	1.4	1.4	0.9 (0.5-1.4)	.57
Wound dehiscence	0.3	0.7	0.3 (0.1-1.1)	.07
Periprosthetic fracture	0.0	0.1	N/A	.99
Aseptic loosening	0.1	0.2	0.3 (0.0-2.6)	.29
90-D medical complications				
Cardiac arrest	0.0	0.1	N/A	.99
Stroke	0.4	0.2	2.3 (0.7-7.2)	.15
Pneumonia	0.8	1.0	0.6 (0.3-1.2)	.14
Deep vein thrombosis	0.6	1.3	0.4 (0.2-0.8)	**.01**
Urinary tract infection	4.8	3.9	1.0 (0.8-1.4)	.79
Acute kidney injury	1.3	0.9	0.9 (0.5-1.6)	.75
*Clostridium difficile* infection	0.2	0.1	2.2 (0.5-10.5)	.31
Hypoglycemic events	1.7	0.4	2.4 (1.4-4.2)	**.001**
Pulmonary aspiration	0.0	0.1	N/A	.99
Resource utilization				
90-D readmission	4.3	3.9	1.0 (0.8-1.4)	.84
Extended LOS (≥3 d)	15.1	22.5	0.5 (0.4-0.6)	**<.001**
1 Y outcomes				
SSI	2.6	1.9	1.0 (0.6-1.4)	.83
Wound dehiscence	0.6	0.9	0.7 (0.3-1.4)	.28
PJI	1.6	1.9	0.7 (0.4-1.1)	.14
Periprosthetic fracture	0.0	0.1	N/A	.99
Aseptic loosening	0.5	0.9	0.4 (0.2-0.9)	**.04**

Bold values indicates significance *P* < .05.

N/A, not available.

a Values are given as the OR with no GLP-1 agonist as the reference group and with the 95% confidence interval (95% CI) in parentheses.

From the matched cohorts (Table 4), there were 1018 patients who were on GLP-1 agonists and 4493 who were not. Multivariable analyses demonstrated no significant differences in 90-day surgical complications. The GLP-1 agonist group had no significant difference in rates of hypoglycemic events after UKA compared with those not on GLP-1 agonists (1.6% vs 0.6%; OR 1.8; *P* = .09). There were no other significant differences in 90-day medical complications. The GLP-1 agonist group also had significantly lower rates of extended LOS (17.7% vs 26.9%; OR 0.6; *P* < .001). Furthermore, at the 1-year mark, patients in the treatment group had no significant difference in rates of SSI (2.3% vs 2.8%; OR 0.8; *P* = .55), PJI (1.3% vs 2.0%; OR 0.6; *P* = .66), or aseptic loosening compared with the control group (0.5% vs 0.9%; OR 0.4; *P* = .11). There were also no differences in rates of pulmonary aspiration (0.0% vs 0.1%; *P* = .99).

**Table 4:** Multivariable analyses of complications—GLP-1 agonist vs no GLP-1 agonist—matched dataset of all patients.

Characteristic	GLP1 agonist (%) N = 1018	No GLP-1 agonist (%) N = 4493	OR^a^ (95% CI)	Multivariable *P*-value
90-D surgical complications				
SSI	1.7	2.1	0.9 (0.4-1.9)	.79
PJI	1.1	1.6	0.7 (0.3-1.6)	.40
Wound dehiscence	0.3	0.7	0.4 (0.2-1.5)	.18
Periprosthetic fracture	0.0	0.1	N/A	.99
Aseptic loosening	0.1	0.3	0.4 (0.0-3.1)	.37
90-D medical complications				
Cardiac arrest	0.0	0.1	N/A	.99
Stroke	0.5	0.2	2.7 (0.9-8.5)	.08
Pneumonia	0.8	1.2	0.7 (0.3-1.5)	.38
Deep vein thrombosis	0.7	1.3	0.5 (0.2-1.1)	.08
Urinary tract infection	4.7	4.2	1.1 (0.7-1.7)	.83
Acute kidney injury	1.5	1.4	0.8 (0.4-1.9)	.69
*Clostridium difficile* infection	0.2	0.1	1.2 (0.1-11.2)	.84
Hypoglycemic events	1.6	0.6	1.8 (0.9-3.5)	.09
Pulmonary aspiration	0.0	0.1	N/A	.99
Resource utilization				
90-D readmission	4.6	4.7	1.1 (0.8-1.5)	.61
Extended LOS (≥3 d)	17.7	26.9	0.6 (0.5-0.7)	**<.001**
1 Y outcomes				
SSI	2.3	2.8	0.8 (0.4-1.6)	.55
Wound dehiscence	0.7	0.8	0.8 (0.4-2.0)	.70
PJI	1.3	2.0	0.6 (0.3-1.4)	.66
Periprosthetic fracture	0.0	0.3	N/A	.99
Aseptic loosening	0.5	0.9	0.4 (0.2-1.2)	.11

Bold values indicates significance *P* < .05.

N/A, not available.

a Values are given as the OR with no GLP-1 agonist as the reference group and with the 95% confidence interval (95% CI) in parentheses.

## Discussion

While several studies have investigated the effects of GLP-1 agonists after primary total hip arthroplasty (THA) and TKA, to the authors’ knowledge, there are no available data regarding GLP-1 agonists and outcomes after UKA [21,22]. This investigation reports that after controlling for patient comorbidities, GLP-1 use was associated with decreased extended LOS after UKA. Notably, our study did not identify increased risk of adverse surgical complications, including PJI, pulmonary aspiration, aseptic loosening, wound dehiscence, SSI, and 90-day readmission after propensity-score matching. These results were similarly demonstrated across TKA studies, supporting the potential low-risk profile of perioperative GLP-1 agonists in patients undergoing UKA [21].

Our study’s demonstration of decreased extended LOS for patients on GLP-1 agonists has been similarly shown in prior studies investigating GLP-1 agonists and their effects after TKA, THA, and anterior cervical discectomy and fusion [21,[35], [36], [37]]. While UKA is generally associated with shorter LOS compared with TKA [38], several benefits are associated with reducing extended LOS, including reduced risks of hospital-acquired infections, increased quality of life, earlier mobilization, and decreased hospital costs [[39], [40], [41]].

Notably, however, patients who were on GLP-1 agonists did have slightly increased rates of hypoglycemia after UKA within our unmatched analysis. This difference was not shown after propensity-score matching, though. Currently, there are limited data about the association of GLP-1 agonists and hypoglycemia within the perioperative period. One randomized controlled trial from Japan found no increased rates of hypoglycemia when combining GLP-1 agonists to sulfonylureas [42]. Similarly, a crossover trial from Denmark looked at 16 patients and identified only 1 episode of hypoglycemia for patients on GLP-1 agonists [43]. As a result, it is possible that the increased rates from our unmatched analysis were related to other patient, anesthesia, or surgical factors. It is also possible that because of the effects of delayed gastric emptying, patients on GLP-1 agonists had a lower food intake compared with patients not on these medications, though further studies would be needed to understand this relationship [44]. Nevertheless, these differences were not shown after appropriate patient covariate matching.

Interestingly, our study identified that patients on GLP-1 agonists were associated with lower rates of 1-year aseptic loosening, though these differences were not significant after appropriate propensity-score matching. In TKA, it is well known that patients who have diabetes are at increased risks for aseptic loosening [45]. In addition, Munger et al. [46] demonstrated that the risk of stem loosening in THA increased by an OR of 1.03 with each additional unit of BMI. Therefore, our findings could potentially be explained by improved glycemic control and weight loss in the treatment group relative to the control. However, this cannot be confirmed as the database does not report changes in patients’ BMI or specific measures of glycemic control, including hemoglobin A1C or point-of-care glucose levels. Despite diagnostic codes for class I/II obesity and class III morbid obesity, as well as complicated vs uncomplicated diabetes being utilized to control for cohorts within the study, the authors acknowledge that without quantitative measures, including BMI, weight, or hemoglobin A1C, bias may potentially be introduced. As a result, our findings are likely correlational, and we cannot conclude that GLP-1 agonists directly decreased rates of aseptic loosening after UKA without further controlled, prospective research.

While the glycemic and weight loss benefits of GLP-1 agonists are well established, as with any medication, GLP-1 agonists are not without potential side effects. In the perioperative state, as previously mentioned, delayed gastric emptying may occur, raising concern for aspiration of gastric contents [44,47]. Previously, the American Society of Anesthesiologists recommended holding GLP-1 agonists 1 week before a patient's scheduled surgery; however, there have been updated collaborative guidelines from the American Society for Metabolic and Bariatric Surgery, American Gastroenterological Association, American Society of Anesthesiologists, International Society of Perioperative Care of Patients with Obesity, and the Society of American Gastrointestinal and Endoscopic Surgeons, which state that patients may continue their GLP-1 agonist therapy before surgery if risks of delayed gastric emptying do not outweigh risks of blood sugar control from discontinuing the GLP-1 agonist, and that patients who are at highest risk for gastrointestinal complications should follow a liquid diet 24 hours prior to their procedure if continuing to take their GLP-1 agonist [48,49]. Our study did not identify any increased risk of 90-day pulmonary aspiration or pneumonia events for patients on GLP-1 agonists. However, it is important to note that the database does not have access to anesthesia, nursing, or operative documentation; therefore, episodes of pulmonary aspiration or regurgitation were reliant on ICD coding, and information regarding the type of anesthesia utilized for surgery was not obtainable.

There are other potential limitations to the study inherent in the use of insurance claims databases. We relied on retrospective examination of administrative data from insurance claims with limited access to clinical data. This methodology, as mentioned previously, is heavily reliant on the accuracy of diagnostic coding to examine comorbidities and outcomes. As such, inaccurate coding could affect the internal validity of the study. Another limitation is the dependence on prescription claim accuracy as a proxy for patient GLP-1 agonist use. While our methodology attempted to identify patients on chronic GLP-1 agonist use perioperatively, we are unable to account for patients using GLP-1 agonists not recorded in the database or those who filled their prescription but did not reliably use it. We are also unable to accurately confirm when patients stopped their medication exactly before surgery. Furthermore, as stated previously, this database does not capture exact BMI, body weight, HbA1c, preoperative and postoperative glucose values, or fructosamine levels; therefore, direct assessment of glycemic control or weight loss—particularly among patients receiving GLP-1 agonists—could not be quantified. As a result, residual confounding and selection bias may influence the results. Prospective studies that incorporate objective metabolic and glycemic measurements are needed to mitigate potential bias and better delineate the impact of GLP-1 agonists on outcomes after UKA.

## Conclusions

This study demonstrated that GLP-1 agonist usage in patients who have T2DM and/or obesity undergoing UKA appears safe from a surgical and medical standpoint and is not associated with adverse complications. In particular, GLP-1 agonists were associated with decreased extended LOS and were not associated with increased rates of PJI, SSI, aseptic loosening, or pulmonary aspiration. Our findings highlight an important potential role of GLP-1 agonists in perioperative medical optimization of diabetic and obese patients undergoing UKA.

## CRediT authorship contribution statement

**Kevin Y. Heo:** Writing – review & editing, Writing – original draft, Visualization, Validation, Software, Methodology, Investigation, Formal analysis, Data curation, Conceptualization. **Jacob A. Worden:** Writing – review & editing, Writing – original draft, Methodology, Investigation, Conceptualization. **Emilio Arellano:** Writing – review & editing, Writing – original draft, Resources, Project administration, Data curation. **Jerry Chang:** Writing – review & editing, Writing – original draft, Visualization. **Bailey J. Ross:** Writing – review & editing, Writing – original draft, Methodology, Conceptualization. **Anthony L. Karzon:** Writing – review & editing, Writing – original draft. **Ajay Premkumar:** Writing – review & editing, Validation, Supervision, Methodology, Conceptualization. **Jacob M. Wilson:** Writing – review & editing, Validation, Supervision, Project administration, Methodology, Investigation, Conceptualization.

## Conflicts of interest

No author associated with this paper has disclosed any potential or pertinent conflicts which may be perceived to have an impending conflict with this work. For full disclosure statements, refer to https://doi.org/10.1016/j.artd.2026.101958.

## Appendix A Supplementary data

Conflict of Interest Statement for Chang

Conflict of Interest Statement for Wilson

Conflict of Interest Statement for Heo

Conflict of Interest Statement for Karzon

Conflict of Interest Statement for Premkumar

Conflict of Interest Statement for Worden

Conflict of Interest Statement for Arellano

Conflict of Interest Statement for Ross
