# Supplementary material for: Outcomes of Unicompartmental Knee Arthroplasty in Patients Receiving Glucagon-like Peptide 1 Agonist Therapy: A Matched Cohort Study
Source: Arthroplast Today. 2026 Feb 9;38:101958. doi: 10.1016/j.artd.2026.101958 (PMC12907661; doi:10.1016/j.artd.2026.101958)
Supplement: Conflict of Interest Statement for Ross [file mmc8.pdf]

INDIVIDUAL CONFLICT OF INTEREST STATEMENT

American Association of Hip and Knee Surgeons

(Adopted from the American Academy of Orthopaedic Surgeons disclosure statement)

The following form **<u>must be filled out completely and submitted by each author (example, 6 authors, 6 forms).</u>**
<u>All items require a response. If there is no relevant disclosure for a given item, enter "*None.*"</u>

Outcomes of Unicompartmental Knee Arthroplasty in Patients Receiving Glucagon-like Peptide 1 Agonist Therapy: A Matched Cohort Study

Manuscript Title

1. Royalties from a company or supplier (The following conflicts were disclosed)

None

2. Speakers bureau/paid presentations for a company or supplier (The following conflicts were disclosed)

None

3A. Paid employee for a company or supplier (The following conflicts were disclosed)

None

3B. Paid consultant for a company or supplier (The following conflicts were disclosed)

None

3C. Unpaid consultants for a company or supplier (The following conflicts were disclosed)

None

4. Stock or stock options in a company or supplier (The following conflicts were disclosed)

None

5. Research support from a company or supplier as a Principal Investigator (The following conflicts were disclosed)

None

6. Other financial or material support from a company or supplier (The following conflicts were disclosed)

None

7. Royalties, financial or material support from publishers (The following conflicts were disclosed)

None

8. Medical/Orthopaedic publications editorial/governing board (The following conflicts were disclosed)

None

9. Board member/committee appointments for a society (The following conflicts were disclosed)

None

<u>Each author must sign AND print or type his/her name, date and submit a separate form</u>

In addition, one BLINDED Conflict of Interest form (no author names used) should be submitted per manuscript with all author disclosures.

Bailey John Ross, MD	[signature]	8/17/25
Author Name (Print or Type)	Author Signature	Date
